# Effectiveness of dry hydrogen peroxide in reducing air and surface bioburden in a multicenter clinical setting

**DOI:** 10.1017/ice.2023.153

**Published:** 2024-04

**Authors:** Don Wright, Jacqueline Christie, Jordan Lawrence, Kimberly L. Vaughn, Timothy F. Walsh

**Affiliations:** 1 Universal Health Services, King of Prussia, Pennsylvania; 2 Department of Public Health, Royal Caribbean Group Health, Miami, Florida; 3 Department of Infection Prevention and Control, Aiken Regional Medical Centers, Aiken, South Carolina; 4 Department of Infection Prevention and Control, Henderson Hospital, Henderson, Nevada; 5 Department of Infection Prevention and Control, Valley Hospital Medical Center, Las Vegas, Nevada

## Abstract

**Objective:**

To determine the effectiveness of dry hydrogen peroxide (DHP) in reducing environmental bioburden in occupied areas.

**Design:**

Prospective environmental cohort study.

**Setting:**

The study was conducted in 2 tertiary-care hospitals and 1 free-standing emergency department.

**Intervention:**

Environmental air and surface sites were cultured before and after continuous deployment of DHP systems in targeted hospital areas.

**Methods:**

In total, 1,554 surface and 1,036 air samples were collected from 74 patient areas among the 3 facilities on 3 consecutive days before DHP deployment and on days 14, 30, 60, and 90 after deployment. At each sampling time, 2 air samples were collected at each facility from 1 room without DHP, along with 2 outdoor samples from each facility. The impact of negative-pressure usage on the efficacy of DHP was also evaluated, with 1 hospital continuously using negative pressure, another utilizing it only in patient isolation scenarios, and another without negative pressure.

**Results:**

In the 2 facilities without continuous negative pressure, exposure to DHP was associated with a significant reduction in surface bioburden, characterized as total colony-forming units (*P* = .019; *P* = .002). Significant associations between DHP exposure and reductions in airborne bacterial load at the 2 hospitals were observed (*P ≤* .001; *P* = .041), and the free-standing emergency department experienced a reduction that did not achieve statistical significance (*P* = .073).

**Conclusions:**

Our findings confirm that DHP has the potential to reduce microbial air and surface bioburden in occupied patient rooms with standard ventilation parameters.

The hospital environment’s role as a potential source of microorganism acquisition and transmission has been well established.^1–4^ Two important factors in the etiology of environmental transmission risk are the extended survival capability of key pathogens in the inanimate environment and the documented inadequacy of standard cleaning and disinfection.^2,5,6^ Research demonstrating the increased infection risk posed by prior room occupants, reported as high as 120%, further underscores the significance of these factors.^7,8^

Automated decontamination technologies have increasingly been utilized to mitigate environmental transmission risk.^8–13^ Numerous studies have shown that their use, as an adjunct to manual cleaning and disinfection, results in greater reductions in environmental microbial bioburden than manual efforts alone.^8–14^

One such technology is dry hydrogen peroxide (DHP). DHP systems contain a 363-nanometer ultraviolet-A bulb used to activate a proprietary photocatalyst applied to a 2-dimensional framed polyester mesh referred to as a “sail.” The technology utilizes ambient humidity and oxygen (ie, no aqueous solution is added or needed) in a series of reactions including photocatalysis and plasma separation to produce DHP, a nonaqueous, near-ideal gas form of the broad-spectrum disinfectant.^15^ The molecules are dispersed throughout a treated space via the room’s air currents, encountering and subsequently oxidizing microbes in the air and on surfaces therein.^15^ DHP can be deployed continuously throughout a treated space, regardless of occupancy status, because the concentrations achieved are well below the acceptable safety limits for human exposure established by the Occupational Safety and Health Administration.^15,16^ DHP starkly contrasts with the aqueous forms of hydrogen peroxide, including vapors or mists, whose use is necessarily restricted to vacant, sealed rooms owing to safety risks posed by their significantly higher concentrations.^2,3^ Additionally, unlike other solutions such as ultraviolet C (UV-C) light, DHP is not adversely affected by shadows but touches all spaces that the air meets. DHP is also distinctly different from bipolar ionization technologies because DHP is a stable molecule that can travel throughout a treated space, whereas bipolar ionization technologies produce a mixture of ionized gas, called plasma, that has a lifespan of <1 second once released from the device before reverting back to the original reagents of humidity and oxygen.^17,18^ Consequently, bipolar ionization’s particulate removal efficacy radically decreases as distance from the ionization device increases due to the decrease in the concentration of surviving ions.^19^

A growing body of evidence in the peer-reviewed literature indicates that DHP effectively reduces microbial bioburden in the occupied healthcare environment.^13,14,20,21^ Studies have additionally shown an association between the use of DHP and a reduction in the incidence of healthcare-acquired infections.^22^ The present multicenter study evaluates the effectiveness of DHP in reducing microbial bioburden in the air and on surfaces, including the effects of negative air pressure, within acute-care facilities.

## Methods

### Setting

This study was conducted in 2 tertiary-care hospitals, Valley Hospital Medical Center (306 beds; hereafter referred to as hospital 1), Aiken Regional Medical Center (273 beds; hereafter referred to as hospital 2) in Nevada and South Carolina, respectively, as well as a freestanding emergency department (hereafter referred to as the FS-ED) in Henderson, Nevada (Table 1).

**Table 1.** Study Sites

Hospital 1	306 acute-care beds, 48-bed behavioral health unit, and 16-bed rehabilitation unit.
Hospital 2	273 acute-care beds, 62-bed behavioral health unit, and 14-bed rehabilitation unit.
Freestanding ER	Emergency room with 11 emergency room beds.

### Facility description

The study was conducted in each facility over 3 different 4-month periods occurring between November 2021 and May 2022. Intervention units included all patient rooms within the intensive care units, intermediate care units, and emergency department of both hospitals along with the surgical intensive care unit of hospital 1. Intervention units within the FS-ED included the patient bays and waiting room. Notably, all intervention patient rooms in hospital 1 continuously operated retrofitted negative-pressure systems, installed as a pandemic response measure, with an estimated 31 air changes per hour (ACH), and with patient room doors closed at all times. Retrofitted negative-pressure controls were continuously used in hospital 2, but only isolation patients had their room doors closed mandatorily, limiting the effective usage of negative pressure to those rooms. Similar to hospital 1, these negative-pressure controls were installed as a pandemic response measure. As defined by the American Society of Heating, Refrigeration, and Air-Conditioning Engineers (ASHRAE), negative-pressure airborne-infectious isolation rooms are designed to bring clean air from the clean zone to the contaminated zone with a minimum pressure differential of 2.5 Pascal (Pa).^23^ To maintain the negative pressure, the exhaust air volume must be at least 10% larger than the supply air volume. The actual negative pressure achieved in a room is influenced by the specific difference in the exhaust air and supply air volumes, airflow paths, and airflow openings (eg, doors, etc).^23^ Accordingly, in hospital 2, negative pressure was only definitively achieved in the rooms in which doors were mandatorily closed (ie, patient isolation rooms). The study protocol was reviewed by institutional review boards for each facility and determined to be exempt (Table 2).

**Table 2.** Variable Directory

Variable Name	Definition
Dry hydrogen peroxide (DHP)	0: Not exposed to DHP at time of collection 1: Exposed to DHP at time of collection
Hospital area (hospital 1)	0: Non-DHP treated area (staff break room) 1: Surgical ICU 2: Intermediate care unit 3: Medical ICU 4: Emergency department
Hospital area (hospital 2)	0: Emergency department 1: Intensive care unit 2: Step-down unit
Outside	Corresponding outdoor airborne CFU count collected at same timepoint
Location_type (hospital 2)	1: Sink counter 2: Bedside table 3: Call button 4: Bedrail
Location_type (FS-ED)	1: Call button 2: Bedrail 3: Sink counter
People	0: Patient room unoccupied during sampling, aside from sampler. 1: Only patient and sampler present during sampling 2: One other person present other than patient and sampler at time of sampling 3: Two or more people present in room during sampling other than patient and sampler
Stool	0: No presence of patient stool near sampling site during collection 1: Presence of patient stool near sampling site during collection
UV_24	0: UV-C technology not used in room within past 24 hours 1: UV-C technology used in room within past 24 hours
Sample_type	0: Bacterial air sample 1: Fungal air sample
Room_type (hospital 2)	0: Patient room 1: Staff room
Room_type (FS-ED)	1: Patient room 2: Waiting room 3: Staff room

Note. ICU, intensive care unit; FS-ED, freestanding emergency department; CFU, colony-forming units; UV-C, ultraviolet C light.

### Environmental sampling

To assess the impact of DHP on environmental bioburden, baseline air and surface sampling prior to the installation of DHP (study days −3, −2, −1) was compared to postimplementation air and surface sampling (study days 14, 30, 60, and 90). All sampling was performed by the respective facility’s infection preventionists.

Surface samples were collected at each timepoint using small Hi-cap swabs containing neutralization buffer (World Bioproducts, Lake County, IL) in 44 of the intervention rooms of hospital 1, 20 intervention rooms of hospital 2, and 10 intervention rooms of the FS-ED. At hospitals 1 and 2, the 3 surface-sampling sites in each room included the bedside table, bed rail, and call button, with the exception of the emergency department in hospital 2, which substituted the sink counter for the bed rail. At the FS-ED, the 3 surface sampling sites in each patient room included the bed rail, call button, and sink counter, and the registration counter and door handle were sampled in the waiting room. Sampling was performed at each timepoint prior to routine, daily cleaning and manual disinfection of the intervention rooms, protocols for which remained unchanged between pre- and postintervention periods at all facilities. Standard cleaning protocols for each facilities are listed as follows:

- Hospital 1: The daily cleaning of the ICU included disinfection of high-touch surfaces in the patient’s room (eg, bedside table, bed rails, door handles) using Oxivir 1 or Virex Plus based on the patient’s isolation status. Terminal cleaning was completed upon patient discharge using the same disinfectants and included UV disinfection.
- Hospital 2: The daily cleaning of high-touch surfaces in the ICU, step-down unit, and emergency department (eg, bed rails, bedside tables, overbed tray tables, door handles, ledges, sink, countertops) included the use of Super-Sani wipes (PDI Healthcare, Woodcliff Lake, NJ) daily. Terminal cleaning was completed upon patient discharge using the same products and included UV disinfection.
- FS-ED: Daily cleaning of the high-touch surfaces (gurney, medical equipment, countertops, doorknobs, light switches, and floors) in these patient rooms, included cleaning with Virex Plus (Diversey, Fort Mill, SC).

The use of UV-C disinfection technologies was tracked at both hospitals and later controlled for using multivariate analysis. At hospital 1, 396 pre-DHP and 528-post DHP surface samples were collected. At hospital 2, 180 pre-DHP and 240 post-DHP surface samples were collected. At the FS-ED, 90 pre-DHP and 120 post-DHP surface samples were collected.

Bacterial and fungal air samples were collected on 90-mm dishes containing tryptic soy agar (bacterial) and Sabouraud dextrose agar (fungal) using an active air sampler (microbial sampler CI-95A, CLiMET, Redlands, CA) in each of the same intervention rooms in which surface samples were collected. Additionally, active bacterial and fungal air sampling was performed in 1 nonpatient room (per facility), such as a break room, without DHP deployment and at 1 outdoor location per facility. At hospital 1, 270 pre-DHP and 360 post-DHP intervention-room air samples were collected. At hospital 2, 120 pre-DHP and 164 post-DHP intervention-room air samples were collected. At the FS-ED, 60 pre-DHP and 80 post-DHP intervention-room air samples were collected. Air sampling for volatile organic compounds (VOCs) was also performed using a VOC meter (Tiger, Ion Science, Fowlmere, Cambridgeshire, UK) in 1 patient room per unit, per facility, per timepoint, with the same room tested each time.

The study team was trained according to collection protocols provided by the third-party laboratory. Sample collection at each facility was supervised by a member of the study team to ensure uniformity of sampling techniques among all 3 study locations.

### Automated DHP systems

The intervention phase of the study involved the deployment of a single standalone DHP unit (Sphere, Synexis, Lenexa, KS) in each of the intervention rooms on study day 0. Each unit was plugged into a standard 120 VAC/220VAC outlet and ran continuously (24 hours a day and 7days a week) throughout the study period. The units generate DHP at a concentration well below the 1 parts per million (ppm) human-exposure safety threshold established by OSHA.^14–16^ All device sails and filters, which are a combination of a Merv 11 and an activated carbon filter, were replaced on day 7 according to the manufacturer’s recommended usage. On day 60, 2 sails and filters from each unit in each facility were inspected to assess the need for changes, and changes were made if indicated. No changes were made to any facility’s existing ventilation parameters.

### Microbiological methods

All samples were shipped overnight to a third-party laboratory for processing (US Microsolutions, Latrobe, PA) with an ice pack to minimize any growth. Surface samples were plated to sheep blood agar plates and were incubated at 20–25°C for 5 days. Air samples were cultured on tryptic soy agar and Sabourad dextrose agar at 20–25°C for bacterial and fungal growth, respectively. Microbial recovery was reported in total colony-forming units (CFU).

### Statistical analysis

To analyze the association between exposure to DHP and microbial load yielded by the collected samples, the CFU counts yielded by the samples were log transformed, and a series of multivariate regression models was created (Stata, College Station, TX). This allowed for the control of covariates that could directly impact microbial load so the primary association of interest could be more accurately assessed. The following covariates were included in the surface sample models: the type of sample location, the area of the hospital, the number of people (not including the sampler) in the room at the time of sampling, the presence of stool in the room during sampling, and if UV-C disinfection had been used in the room within the past 24 hours. For the models evaluating the air samples, the following covariates were included: the type of sample (ie, bacterial or fungal), the type of room (ie, patient room, waiting room, or break room), the number of people present (not including the sampler) in the room at the time of sampling, and the microbial load yielded from the corresponding outdoor sample. An association between 2 variables was considered significant if the corresponding *P* value was ≤.05.

## Results

### Hospital 1

At hospital 1, which continuously employed negative pressure with patient room doors closed, there was no detected association between exposure to DHP and surface microbial load. However, there was a statistically significant relationship between exposure to DHP and reduced airborne bacterial levels (*P ≤* .0001), controlling for the area of the hospital, the type of room, number of people within the room during sampling, and the outdoor airborne bacterial level. Minimal levels of airborne fungal bioburden were detected throughout the course of the entire study, limiting the study team’s ability to evaluate the relationship between DHP and airborne fungal levels at this facility. Additionally, the level of VOCs decreased by 75.1% from the baseline average of 2.53 ppm to a postintervention average of 0.63 ppm. Consistent levels of VOCs were detected in the nonintervention control room throughout the study (0.5 ppm during the pre- and postintervention periods).

### Hospital 2

**Figure 1.**
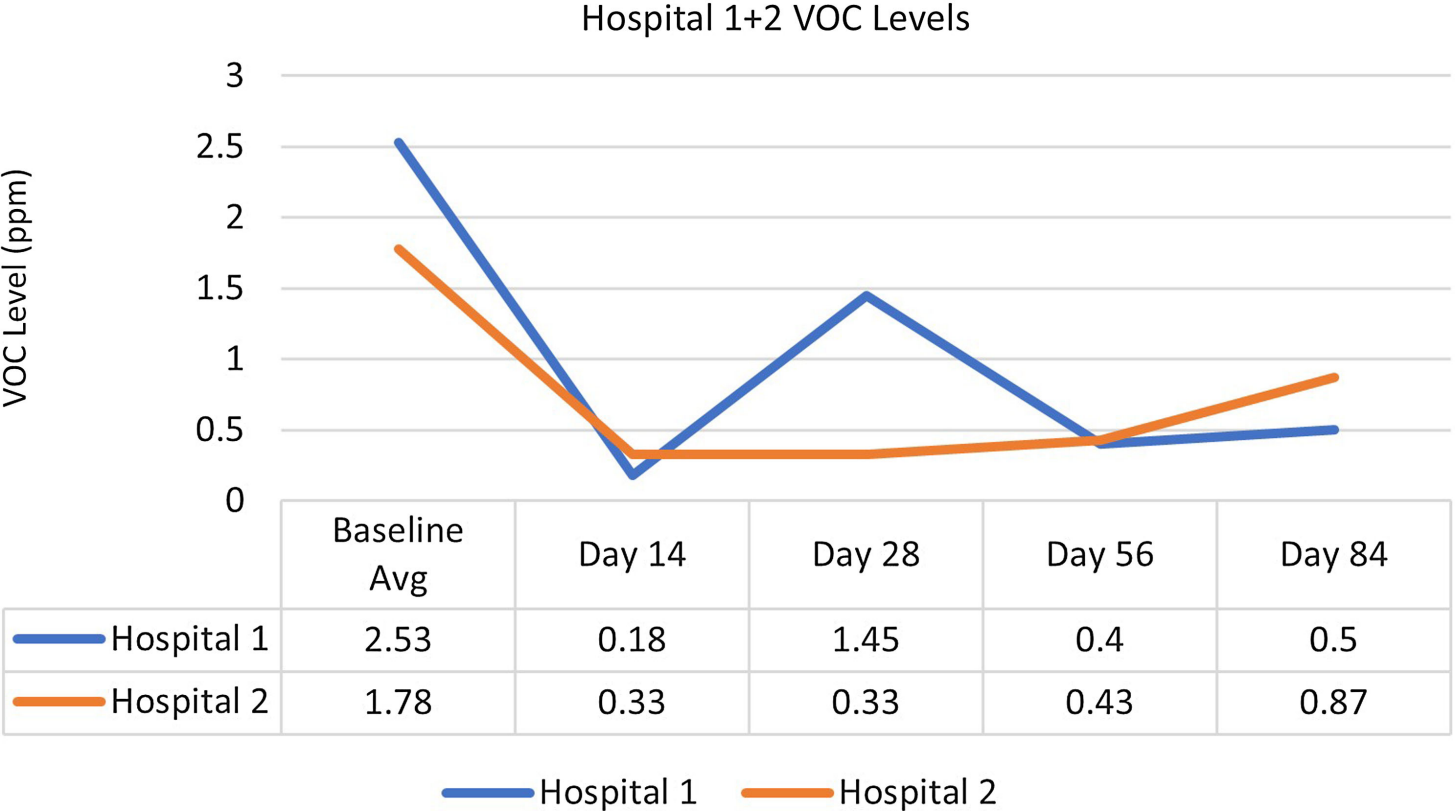
Volatile organic compound (VOC) levels for hospitals 1 and 2.

At hospital 2, which continuously operated negative-pressure systems but only mandatorily closed doors to patient isolation rooms, a statistically significant association was detected between exposure to DHP and a reduction in airborne microbial load, controlling for the sample type, room type, number of people in the room at the time of sampling, and the outdoor airborne fungal and bacterial levels (*P* = .041). Similarly, a statistically significant association was detected between exposure to DHP and a reduction in surface microbial load, controlling for the type of sample location, hospital area, the number of people present during sampling, the presence of stool, and exposure to UV-C disinfection systems within the past 24 hours (*P* = .019). Additionally, the level of VOC decreased by 72.5% from the baseline average of 1.78 ppm to a postintervention average of 0.49 ppm. Conversely, the VOC level in the nonintervention control room doubled from 0.2 ppm at baseline to 0.4 ppm during the postintervention period (Fig. 1).

### Free-standing emergency department

At the free-standing emergency department, where no negative-pressure systems were deployed, a statistically significant association was detected between exposure to DHP and a reduction in surface microbial load, controlling for location type, the number of people present within the room during sampling, and the type of room (*P* = .002). A significant association between DHP exposure and airborne microbial load was not detected, but strong directionality was present (*P* = .073), controlling for room type, the number of people present during sampling, and the corresponding outdoor microbial load. At this facility, VOC levels at baseline and after the intervention consistently remained at 0.1 ppm or below.

## Discussion

These results provide strong evidence that DHP affects surface and airborne microbial load, including both bacterial and fungal in the latter category. The results also indicate that the continuous usage of negative pressure within a patient room affects the ability of DHP to reach all surfaces within the space before ventilation. Hospital 2 and the FS-ED both experienced statistically significant reductions in surface microbial load during the postintervention phase that was significantly associated with DHP exposure when controlling for known covariates. The FS-ED experienced reductions in both airborne bacterial and fungal microbial load over the same period, but statistical significance was not detected at this facility, possibly due to the limited sample size. This hypothesis is supported by the DHP-associated statistically significant reductions in airborne bacterial and fungal microbial load observed at hospital 2, which included a sample size double that of the FS-ED. The universal reductions observed at hospital 2 indicate that DHP can affect both airborne and surface microbial load when negative pressure is employed in selective scenarios, such as isolation patient rooms. The results from hospital 1 indicate that the impact of DHP on microbial load is restricted to airborne bacteria in a setting that employs continuously operating negative-pressure systems with the doors closed.

The consistently low airborne fungal counts in this study limited our ability to evaluate the impact of DHP on airborne fungi at this facility. At both hospital 1 and hospital 2, VOC reductions were observed in the patient rooms during the postintervention phase, providing further evidence of DHP’s airborne impact in both types of negative-pressure settings.

Our findings add to the existing body of knowledge that confirms that DHP has the potential to reduce microbial air and surface bioburden in occupied patient rooms with standard ventilation parameters. Further studies are needed to explore the effect of DHP on space with active negative air-pressure controls with different size parameters and air exchanges. DHP is an environmental disinfection method that may positively affect patient health and safety in healthcare settings.

## Appendix: Regression Models

Model 1: Hospital 1 Airborne Bacterial Load

Model 2: Hospital 2 Surface Microbial Load

Model 3: Hospital 2 Airborne Microbial Load

Model 4: FS-ED Surface Microbial Load

Model 5: FS-ED Airborne Bacterial Load
